# A case-matched study of stereotactic radiosurgery for patients with brain metastases: comparing treatment results for those with versus without neurological symptoms

**DOI:** 10.1007/s11060-016-2264-0

**Published:** 2016-09-03

**Authors:** Takao Koiso, Masaaki Yamamoto, Takuya Kawabe, Shinya Watanabe, Yasunori Sato, Yoshinori Higuchi, Tetsuya Yamamoto, Akira Matsumura, Hidetoshi Kasuya, Bierta E. Barfod

**Affiliations:** 1 Katsuta Hospital Mito GammaHouse, 5125-2 Nakane, Hitachi-naka, Ibaraki 312-0011 Japan; 2 Department of Neurosurgery, Faculty of Medicine, University of Tsukuba, 2-1-1 Amakubo, Tsukuba, Ibaraki 305-8576 Japan; 3 Department of Neurosurgery, Tokyo Women’s Medical University Medical Center East, 8-1-10 Nishiogu, Arakawa-ku, Tokyo, 104-0045 Japan; 4 Department of Neurosurgery, Kyoto Prefectural University of Medicine, Graduate School of Medical Sciences, 465 Kawaramachi-Hirokoji, Kamigyo-ku, Kyoto, 602-8566 Japan; 5 Department of Neurosurgery, National Hospital Organization Mito Medical Center, 280 Sakuranosato, Ibaraki-machi, Ibaraki 311-3193 Japan; 6 Clinical Research Center, Chiba University Graduate School of Medicine, 1-8-1 Inohana, Chuo-ku, Chiba, 260-8677 Japan; 7 Department of Neurological Surgery, Chiba University Graduate School of Medicine, 1-8-1 Inohana, Chuo-ku, Chiba, 260-8677 Japan

**Keywords:** Brain metastases, Radiation therapy, Radiosurgery, Gamma knife, Metastatic tumor

## Abstract

We aimed to reappraise whether post-stereotactic radiosurgery (SRS) results for brain metastases differ between patients with and without neurological symptoms. This was an institutional review board-approved, retrospective cohort study using our prospectively accumulated database including 2825 consecutive BM patients undergoing gamma knife SRS alone during the 15-year period since July 1998. The 2825 patients were divided into two groups; neurologically asymptomatic [group A, 1374 patients (48.6 %)] and neurologically symptomatic [group B, 1451 (51.4 %)]. Because there was considerable bias in pre-SRS clinical factors between groups A and B, a case-matched study was conducted. Ultimately, 1644 patients (822 in each group) were selected. The standard Kaplan–Meier method was used to determine post-SRS survival. Competing risk analysis was applied to estimate cumulative incidences of neurological death, neurological deterioration, local recurrence, re-SRS for new lesions and SRS-induced complications. Post-SRS median survival times (MSTs) did not differ between the two groups; 7.8 months in group A versus 7.4 months in group B patients (HR 1.064, 95 % CI 0.963–1.177, p = 0.22). However, cumulative incidences of neurological death (HR 1.637, 95 % CI 1.174–2.281, p = 0.0036) and neurological deterioration (HR 1.425, 95 % CI 1.073–1.894, p = 0.014) were significantly lower in the group A than in the group B patients. Neurologically asymptomatic patients undergoing SRS for BM had better results than symptomatic patients in terms of both maintenance of good neurological state and prolonged neurological survival. Thus, we conclude that screening computed tomography/magnetic resonance imaging is highly beneficial for managing cancer patients.

## Introduction

Brain metastases (BMs) are generally a life-threatening neurological problem for cancer patients regardless of primary tumor sites [1–4]. However, due mainly to recent advances in systemic cancer treatment [5, 6], an appropriately selected BM patient subgroup can now achieve longer survival with maintenance of good neurological function if BMs are well controlled. It is now widely recognized that not only prolonged survival but also maintenance of good neurological function is crucial for managing patients with BMs. Thus, successful management requires precise BM diagnosis in an early disease stage, i.e., before a BM manifests with neurological symptoms. Figlin et al. reported BM to be discovered within 2 years after initial treatment of lung cancer [7]. Moreover, with widespread use of computed tomography (CT) and/or magnetic resonance images (MRI), increasing numbers of patients with asymptomatic BMs are being diagnosed [5, 6, 8–10]. Most notably, MRI is more sensitive than CT for BM detection [5, 10–14]. BMs were reportedly found in 6–30 % of initially-diagnosed lung cancer patients [6, 10, 15–19] and were neurologically silent in 14 % [5].

As Lippitz et al. recently reviewed extensively, stereotactic radiosurgery (SRS) has become an established treatment option for managing BM patients [20]. SRS is more advantageous than other treatments, i.e., whole brain radiotherapy (WBRT), surgery, systemic anti-cancer agent therapy and combinations of these modalities, in terms of costs, hospitalization, morbidity, mortality and wider applicability and repeatability [21]. With all cancer treatment modalities, it is generally believed that both early diagnosis and immediate treatment improve patient outcomes. Although considerable numbers of series, both prospective and retrospective, have demonstrated treatment results including neurological survival and maintenance of neurological condition in BM patients undergoing SRS, little is known about whether post-SRS results for BM differ between patients with and without neurological symptoms. We aimed to reappraise whether neurologically-asymptomatic patients are more likely to benefit from SRS, in terms of both neurological survival and maintenance of neurological condition, than symptomatic patients.

## Materials and methods

### Patient population

This was an institutional review board (IRB)-approved, retrospective cohort study using our prospectively accumulated database including 2825 consecutive BM patients undergoing gamma knife SRS alone during the 15-year period between July 1998 and June 2013 (Tokyo Women’s Medical University IRB: #1981). As all patients had been referred to us for SRS, their primary physicians had mostly made the patient selections. Patient selection criteria may thus have differed among referring physicians. Therefore, one author (MY) decided whether or not to accept a patient. We did not perform SRS on patients with low Karnofsky Performance Status (KPS) scores (<70 %) due to systemic diseases, a non-cooperative state due to poor neurocognitive function, meningeal dissemination, or an anticipated survival period of 3 months or less. As to tumor size, if MRI demonstrated tumors with diameters of 2–3 mm in the brainstem or the optic apparatus, we performed SRS rather than further follow-up observation. Otherwise, SRS was usually postponed with close MRI follow-up until the tumor diameter exceeded approximately 1 cm. Table 1 summarizes clinical characteristics, overall and for the two groups, i.e. neurologically asymptomatic (group A, 1374 patients) and symptomatic (group B, 1451 patients). As mentioned above, the primary physicians responsible for each patient decided the indications for both surgery and radiotherapy. Therefore, prior to SRS, among the 2825 patients, 523 (18.5 %) had undergone surgical removal of brain METs and 141 (4.8 %) WBRT (Table 1).

**Table 1:** Summary of clinical characteristics of 2825 brain metastasis patients (cohort)

Characteristics	Total	Neurological symptoms		p values^a^
		No (group A)	Yes (group B)	
No. of patients	2825	1374	1451	
Age (years)				
Mean	64	64	65	0.05
Range	19–96	19–96	19–93	
Sex				
Female	1134 (40.1 %)	547 (39.8 %)	587 (40.5 %)	0.73
Tumor numbers				
Mean	7	7	7	0.45
Median	3	3	3	
Range	1–89	1–69	1–89	
IQR	1–8	1–8	1–8	
Primary cancer sites				
Lung	1840 (65.1 %)	1046 (76.1 %)	794 (54.7 %)	<0.001^b^
Breast	309 (10.9 %)	122 (8.9 %)	187 (12.9 %)	
GI tract	328 (11.6 %)	87 (6.3 %)	241 (6.9 %)	
Kidney	115 (4.1 %)	47 (3.4 %)	68 (3.3 %)	
Others	233 (5.7 %)	72 (5.2 %)	161 (11.1 %)	
Primary cancer status				
Controlled	865 (30.7 %)	414 (30.1 %)	451 (31.1 %)	0.60
Extra-cerebral METs				
No	1456 (51.5 %)	695 (50.6 %)	679 (49.4 %)	0.33
KPS				
≥80 %	2150 (76.1 %)	1176 (85.6 %)	974 (67.1 %)	<0.001
Modified-RPA class [34, 35]				
1 + 2a	638 (22.6 %)	363 (26.4 %)	275 (19.0 %)	
2b	872 (30.9 %)	457 (33.3 %)	415 (28.6 %)	0.08^c^
2c + 3	1315 (46.5 %)	554 (40.3 %)	761 (52.5 %)	<0.001^d^
Prior surgery				
Yes	523 (18.5 %)	113 (8.2 %)	410 (28.3 %)	<0.001
Prior WBRT				
Yes	141 (5.0 %)	85 (6.2 %)	56 (3.9 %)	0.006
Tumor volume (cc)				
Cumulative				
Mean	9.74	6.43	12.87	<0.001
Range	0.01–126.2	0.01–115.3	0.04–126.2	
IQR	1.89–12.92	0.89–8.21	3.69–17.45	
Largest tumor				
Mean	6.78	4.42	9.02	<0.001
Range	0.01–94.20	0.01–94.20	0.02–89.30	
IQR	1.10–8.81	0.52–5.22	2.60–11.80	
Peripheral dose (Gy)				
Mean	21.12	22.01	20.52	<0.001
Range	10.00–32.00	12.00–32.00	10.00–25.00	
IQR	20.00–24.00	20.00–24.00	18.00–24.00	

*IQR* interquartile range, *GI* gastrointestinal, *METs* metastases, *KPS* Karnofsky Performance Status,^*12*^ *RPA* recursive partitioning analysis, ^*4*^ *WBRT* whole brain radiotherapy, *CI* confidence interval

^a^Student *t* test was used for continuous variables and Fisher’s exact test for pairs of categorical variables, ^b^Lung versus non-lung, ^c^Modified-RPA classes 1 + 2a versus 2b, ^d^Modified-RPA classes 2b versus 2c + 3

Before SRS, the treatment strategies were explained in detail to each patient, as well as at least one adult relative, and written informed consent was obtained from all patients before SRS by the second author (M.Y.). As our previous report described our radiosurgical techniques in detail, they are not repeated herein [22, 23]. Briefly, standard SRS procedures were performed using a Leksell gamma unit Model B before June, 2003, and thereafter a Leksell gamma unit Model C (Elekta AB, Stockholm, Sweden).

Post-SRS, all patients were routinely managed by referring physicians and were recommended to have clinical and neuro-imaging examinations at an approximately 2–3 month interval. Neuroimaging follow-up could not be performed in 815 patients (28.9 %); mostly because of post-SRS early deterioration due to systemic diseases [median survival time of this subset was 2.5 (95 % CI 2.3–2.6 months)]. Approximately 50 % of our 2825 patients came to our outpatient clinic periodically, while clinical and/or neuroimaging data were sent to us by post in about 25 % of cases. The second author (M.Y.) called the remaining 25 % of patients or their relatives by telephone to confirm the patients’ conditions. For deceased patients, the day of death, cause of death, and detailed information on patient condition changes were requested by telephone.

### Clinical outcomes

As clinical outcomes were described in detail in our previous report, they are not repeated herein [22, 23]. Briefly, the primary endpoint was overall survival, and the secondary endpoints were neurological death, neurological deterioration, local recurrence of the treated tumor, repeat SRS for new lesions, salvage WBRT, salvage surgery, and SRS-induced major complications. For each endpoint, failures were regarded as events and any others as censored. Overall survival time was defined as the interval between the first SRS and death due to any cause (progression of systemic metastases and/or BM, other disease unrelated to cancer, accident, suicide, and so on) or the day of the last follow-up. Neurological death was defined as death caused by any intracranial disease, including tumor recurrence, carcinomatous meningitis, cerebral dissemination, and progression of other untreated intracranial tumors. Neurological deterioration-free survival time was defined as the interval between the first SRS and the day that any brain disease-caused neurological worsening manifested (that is, local recurrence, progression of new lesions, and SRS-induced complications). Decreases in KPS scores, in patients with scores of 20 % or less, due to neurological worsening were regarded as events and any others as censored. Major complication-free survival time was taken as the interval between the first SRS and the day major SRS-induced complications occurred. Patients with major complications included those with Radiation Therapy Oncology Group (RTOG) neurotoxicity grades of 2 or worse and, even if the grade was either 0 or 1, those in whom surgical intervention was required based on sequential MRI follow-up demonstrating progressive enlargement of a cyst and/or a mass lesion with further observation thus being regarded as excessively high risk; all of these conditions were regarded as events and any others as censored [24].

### Statistical analysis

All data were analyzed according to the intention-to-treat principle. For baseline variables, summary statistics were constructed using frequencies and proportions for categorical data and medians, ranges and interquartile ranges (IQR) for continuous variables. We compared patient characteristics using the Fisher exact test for categorical outcomes and t-tests for continuous variables, as appropriate. The Kaplan–Meier method was used to estimate overall survival. Moreover, univariate analysis using the Cox proportional hazard model was performed to determine pre-SRS clinical factors favoring longer survival.

For time-to-event outcomes, the cumulative incidences of neurological death, neurological deterioration, local recurrence, repeat SRS, and major complications were estimated by a competing risk analysis, because death is a competing risk for loss to follow-up (that is, patients who die can no longer become lost to follow-up) [25–27]. Also, to identify baseline and clinical variables associated with the 5 aforementioned outcomes, competing risk analyses were performed with the Fine–Gray generalization of the proportional hazards model accounting for death as a competing risk [28]. Fine–Gray generalization makes use of the sub-distribution hazard to model cumulative incidence, thereby quantifying the overall benefit or harm of an exposure [29].

All comparisons were planned and the tests were two-sided. A p value of <0.05 was considered to be statistically significant. All statistical analyses were performed by a statistician (YS) using SAS software version 9.4 (SAS Institute, Cary, NC, USA) and the R statistical program, version 3.10. Before statistical analyses, the database was cleaned (by YH). These two authors were not involved in either SRS treatment or patient follow-up.

## Results

### Cohort studies

**Fig. 1:**
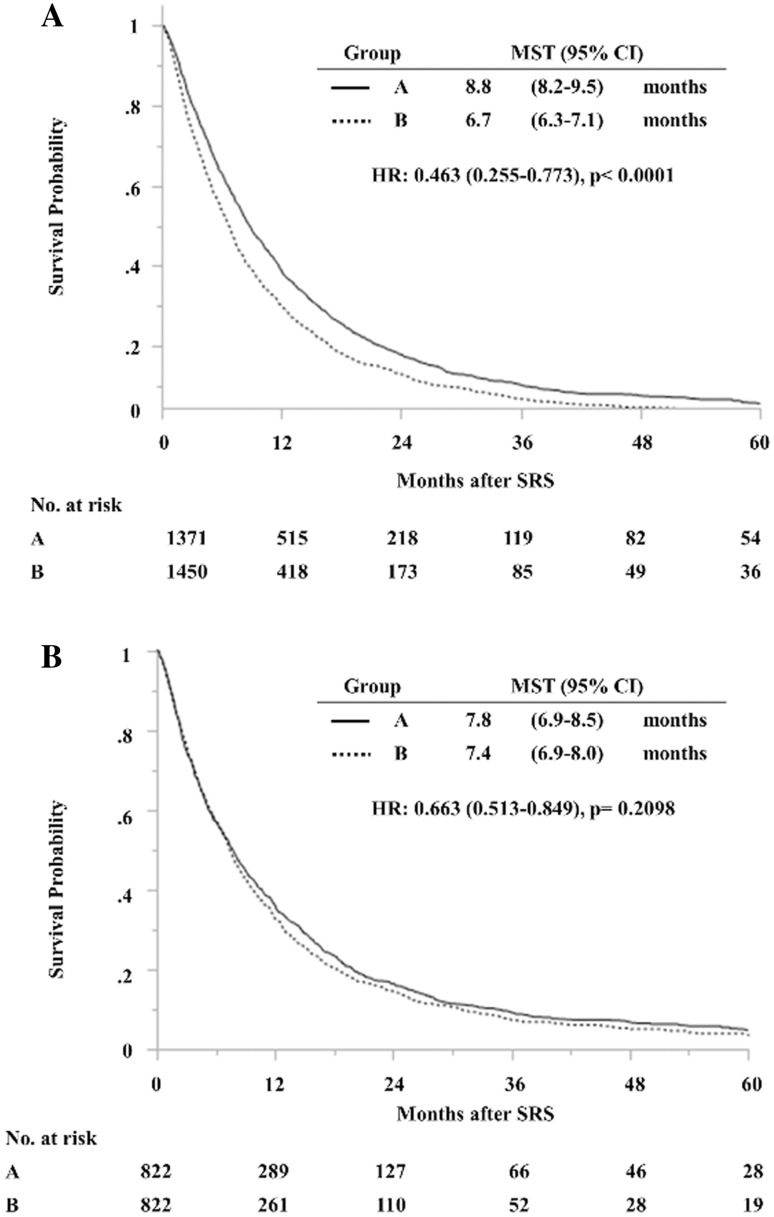
Overall survival based on 2825 patients (**a**) and on a subset of 1644 case-matched patients (**b**) according to neurological symptoms [asymptomatic (group A) and symptomatic (group B)], estimated using the standard Kaplan–Meier method

Four patients were lost to follow-up (0.34 %, three in group A and one in group B). As of January 2014, the median post-SRS observation time among censored observations (212 patients) was 41.9 (IQR 8.7–54.0, maximum 173.5) months and 2609 patients (92.4 %) had died. The overall median survival time (MST) after SRS was 7.6 months (95 % CI 7.3–8.0 months). Actuarial post-SRS survival rates were 58.6, 34.3, 15.4, 8.6, and 4.7 % at the 6th, 12th, 24th, 36th, and 60th post-SRS month, respectively. Among the 2609 deceased patients, causes of death could not be determined in 109, but were confirmed in the remaining 2500 to be non-brain disease in 2211 (88.4 %) and brain disease in 289 (11.6 %). MST after SRS was significantly longer in the 1374 group A (8.9 months) than in the 1451 group B (6.7 months) patients (HR 1.238, 95 % CI 1.146–1.337, p < 0.001) (Fig. 1).

Among 1840 patients with lung cancer, BMs had not manifested with neurological symptoms at the time of SRS in 1046 (56.9 %). However, 39.5 % (122/309 patients) of breast cancer, 26.5 % (87/328) of gastro-intestinal tract cancer and 40.9 % (47/115) of renal cancer patients underwent SRS for asymptomatic BMs.

### Studies of case-matched subset

As shown in Table 1, proportions of patients with some of the clinical characteristics differed significantly between groups A and B, i.e., proportions of primary cancer sites, KPS, Modified-RPA classes, and prior treatments (surgery and WBRT) were strikingly uneven and both cumulative tumor volumes and the volumes of the largest tumors differed significantly between the two groups. These differences might have impacted survival and/or neurological deterioration. Therefore, a case-matched study was conducted by one of the authors (Y.S.), who did not participate in other aspects of this study and was blinded to final outcomes. Patient selection was performed employing the propensity score matching method with a Greedy 5-To-1 Digit-Matching algorithm [30] for clinical factors (primary tumor sites, Karnofsky score, Modified-Recursive Partitioning Analysis classes, prior procedures [surgery and WBRT], cumulative tumor volume, volume of the largest tumor, and peripheral doses), all of which had p values <0.05 [31, 32]. After all of the propensity-score matches had been performed, we compared baseline characteristics between the two groups. Ultimately, 1644 patients (822 in each group) were selected. The p values after matching were over 0.05 for all clinical factors except age (Table 2). However, the difference in mean ages was only 1.2 years (63.8 years/group A vs. 65.0 years/group B). This difference is not considered to be clinically meaningful.

**Table 2:** Summary of clinical characteristics of 1644 case-matched brain metastasis patients

Characteristics	Total	Neurological symptoms		p values^a^
		No (group A)	Yes (group B)	
No. of patients	1644	822	822	
Age (years)				
Mean	64	64	65	0.04
Range	19–96	19–96	19–93	
Sex				
Female	650 (39.5 %)	327 (39.8 %)	323 (39.3 %)	0.88
Tumor numbers				
Mean	7	8	7	0.87
Median	3	4	3	
Range	1–89	1–63	1–74	
IQR	1–9	1–9	1–8	
Primary cancer sites				
Lung	1077 (65.5 %)	531 (64.6 %)	546 (66.4 %)	0.47^a^
Breast	192 (11.7 %)	101 (12.3 %)	91 (11.1 %)	
GI tract	172 (10.5 %)	87 (10.6 %)	85 (10.3 %)	
Kidney	79 (4.8 %)	42 (5.1 %)	37 (4.5 %)	
Others	124 (7.5 %)	61 (7.4 %)	63 (7.7 %)	
Primary cancer status				
Controlled	507 (30.8 %)	254 (30.9 %)	253 (30.8 %)	1.00
Extra-cerebral METs				
No	830 (50.5 %)	412 (50.1 %)	418 (50.9 %)	0.81
KPS				
≥80 %	1291 (78.5 %)	647 (78.7 %)	644 (78.4 %)	0.90
Modified-RPA class [34, 35]				
1 + 2a	354 (21.5 %)	172 (20.9 %)	182 (22.1 %)	
2b	528 (32.1 %)	272 (33.1 %)	256 (31.1 %)	0.41^c^
2c + 3	762 (46.4 %)	378 (46.0 %)	384 (46.7 %)	0.53^d^
Prior surgery				
Yes	231 (14.1 %)	113 (13.8 %)	118 (14.4 %)	0.78
Prior WBRT				
Yes	75 (4.6 %)	36 (4.4 %)	39 (4.7 %)	0.81
Tumor volume (cc)				
Cumulative				
Mean	8.92	8.80	9.04	0.87
Range	0.01–115.3	0.01–115.3	0.05–77.88	
IQR	2.18–11.74	1.80-11.66	2.62–11.95	
Largest tumor				
Mean	6.17	6.06	6.29	0.65
Range	0.02–94.20	0.02–94.20	0.05-70.00	
IQR	1.31–7.80	0.92–7.82	1.80–7.80	
Peripheral dose (Gy)				
Mean	21.32	22.28	21.30	0.55
Range	10.00–25.00	12.00–32.00	12.00–25.00	
IQR	20.00–24.00	20.00–24.00	20.00–24.00	

*IQR* interquartile range, *GI* gastrointestinal, *METs* metastases, *KPS* Karnofsky Performance Status,^*12*^ *RPA* recursive partitioning analysis, ^*4*^ *WBRT* whole brain radiotherapy, *CI* confidence interval

^a^Student *t* test was used for continuous variables and Fisher’s exact test for pairs of categorical variables, ^b^Lung versus non-lung, ^c^Modified-RPA classes 1 + 2a versus 2b, ^d^Modified-RPA classes 2b versus 2c + 3

As shown in Fig. 1, although the post-SRS MST was slightly longer in group A (7.8 months) than in group B (7.4 months) patients, this difference was not statistically significant (HR 1.064, 95 % CI 0.963–1.177, p = 0.22). Both crude and cumulative incidences of neurological death were significantly lower in group A than in the group B patients (HR 1.637, 95 % CI 1.174–2.281, p = 0.0036) (Tables 3, 4). Although crude incidences of neurological deterioration did not differ significantly between the two patient groups, cumulative incidences of neurological deterioration were significantly lower in the group A than in the group B patients (HR 1.425, 95 % CI 1.073–1.894, p = 0.014).

**Table 3:** Summary of treatment results after stereotactic radiosurgery (SRS)

	Neurological symptoms		p value
	No (group A)	Yes (group B)	
No. of patients	822	822	
Neurological death^a^	58 (7.9 %)	88 (11.9 %)	0.01
Neurological deterioration	84 (10.2 %)	110 (13.4 %)	0.06
Local recurrence^b^	48 (8.5 %)	57 (9.5 %)	0.54
Repeat SRS	259 (31.5 %)	224 (27.3 %)	0.07
Salvage WBRT	36 (4.4 %)	23 (2.8 %)	0.11
Salvage surgery	14 (1.7 %)	15 (1.8 %)	1.00
SRS-related complications	30 (3.7 %)	24 (2.9 %)	0.49

^a^Based on 1471 [734 (96.0 %) in group A and 737 (96.6 %) in group B, p = 0.59] deceased patients whose causes of death were determined (57 patients were excluded because causes of death were not available)

^b^Based on 1165 [567 (69.0 %) in group A and 598 (72.8 %) in group B, p = 0.10] patients (479 patients were excluded because neuro-imaging results were not available)

**Table 4:** Summary of time-to-event outcome studies using competing risk analyses

	Cumulative incidences (post-SRS months)				HR (95 % CI)	p value
	6	12	24	36		
Neurological death					1.637 (1.174–2.281)	0.0036
Asymptomatic (group A)	0.021	0.033	0.057	0.068		
Symptomatic (group B)	0.030	0.073	0.100	0.110		
Neurological deterioration					1.425 (1.073–1.894)	0.014
Asymptomatic (group A)	0.032	0.057	0.080	0.096		
Symptomatic (group B)	0.047	0.093	0.121	0.130		
Local recurrence^a^					1.360 (0.926–1.998)	0.11
Asymptomatic (group A)	0.017	0.046	0.062	0.072		
Symptomatic (group B)	0.023	0.057	0.091	0.099		
Repeat SRS					0.880 (0.736–1.053)	0.16
Asymptomatic (group A)	0.153	0.256	0.301	0.318		
Symptomatic (group B)	0.131	0.229	0.269	0.275		
SRS-related complications					0.088 (0.514–1.507)	0.64
Asymptomatic (group A)	0.006	0.013	0.021	0.026		
Symptomatic (group B)	0.012	0.021	0.027	0.027		

*SRS* stereotactic radiosurgery, *HR* hazard ratio, *CI* confidence interval

^a^Based on 1165 [567 (69.0 %) in group A and 598 (72.8 %) in group B, p = 0.10] patients (479 patients were excluded because neuro-imaging results were not available)

Crude and cumulative incidences of local recurrence, repeat-SRS and SRS-related complications did not differ significantly between the two patient groups, as shown in Tables 3 and 4. Also, the crude incidence of salvage treatment, i.e., surgery or WBRT, did not differ significantly between the two patient groups (Table 3).

### Four major original cancers

There were no significant MST differences between the two groups, A and B, in patients with lung, gastro-intestinal and renal cancers (Table 3). However, in those with breast cancer, the MST of the group A patients [12.3 (95 % CI 8.7–16.0) months] was significantly longer than that of the group B patients [8.3 (6.4–10.8) months, p = 0.01]. In lung cancer patients, the crude incidence of neurological death was significantly higher in the group B patients (12.8 %) than in the group A patients (8.1 %, p = 0.02). However, there were no significant differences in the incidence of neurological death between the A and B groups for patients with breast, gastro-intestinal and renal cancers. Also, in lung cancer patients, the crude incidence of neurological deterioration in the group B patients (15.2 %) was significantly higher than that in the group A patients (10.0 %, p = 0.01). There were, however, no significant differences in the incidence of neurological deterioration between groups A and B for patients with breast, gastro-intestinal and renal cancers.

## Discussion

Debate continues as to whether post-treatment survival periods are significantly longer in neurologically asymptomatic patients than in symptomatic patients. Neurologically asymptomatic patients reportedly have better outcomes [2, 33–36], though some authors have argued against such a relationship [6, 17, 37]. However, all previously reported series were based on relatively small sample sizes. Furthermore, only overall survivals were discussed in prior reports, with neithor neurological survival nor maintenance of neurological condition being described. Maintenance of good neurological function and, eventually, a decreased incidence of neurological death, have recently been recognized as being crucial for managing patients with BMs. We consider our herein-reported dataset, with a relatively large sample size, to show that SRS, prior to BMs becoming neurologically symptomatic, has the potential to achieve prolonged maintenance of neurological function and to minimize neurological death, although MST did not differ significantly between the two groups.

In general, earlier diagnosis and non-delayed treatment are believed to improve treatment outcomes for patients with all types of malignant tumors. However, based on 181 patients, Seute et al. reported chemotherapies to be ineffective for asymptomatic BM from small cell lung cancer [17]. Kim et al. also reported that there was no significant difference in overall survivals between 7 asymptomatic and 31 symptomatic patients with BM from non small cell lung cancer (NSCLC) (43 vs. 45 weeks, p = 0.3689) [6]. Kuba et al. obtained similar results in 53 asymptomatic and 12 symptomatic patients with BM from breast cancer (12 vs. 13 months, p = 0.99) [37]. Brain CT for lung cancer patients is recommended only for those who are neurologically symptomatic, because screening neuro-imaging examinations are not cost-effective for asymptomatic patients [38, 39]. We disagree with this view. We advocate considering that once a cancer patient becomes neurologically-handicapped, medical costs far exceed that for a single neuro-imaging examination using either CT or MRI, though it must be noted that these costs vary markedly among countries, i.e., approximately US$300 in Japan, even if a contrast study is added, and US$3000 in the USA. Moreover, expenses are not the only consideration. The difficulty and anxiety experienced by neurologically-handicapped patients must also be taken into account. In fact, most physicians in Japan are now performing screening MRI for lung cancer patients, even those who are neurologically asymptomatic, as described above. We advocate that this policy be generally adopted by physicians who manage cancer patients, regardless of the original tumor type.

However, it should be kept in mind that BMs occur at a relatively early disease stage in lung cancer patients as compared to those with breast, gastro-intestinal tract or kidney cancers, as shown in Table 5 (on line only). This issue makes it difficult to perform screening MR imaging examinations for all patients with non-lung cancers. In fact, the high costs of neuro-imaging examinations are considered to be a major hurdle to routinely using CT/MRI for cancer patients without neurological symptoms in the USA. Nevertheless, as described above, SRS for non-symptomatic BMs was clearly shown to have survival benefits in breast cancer patients. As with lung cancer patients, we recommend screening neuro-imaging examinations for breast cancer patients at high risk for BMs, i.e., as reported by Barnholtz-Sloan et al. and Shouteen et al. who found incidences of BMs to be higher in patients with advanced disease [40, 41]. In this study, we found that performing SRS for non-symptomatic BMs in patients with gastro-intestinal and renal cancers provided no benefits in terms of neurological death or maintenance of neurological condition. For these patients, periodic CT examinations for re-staging consistently including the brain are worthwhile for detecting BM before they manifest with neurological symptoms.

**Table 5:** Number of patients with synchronous versus metachronous presentation and interval between diagnosis of primary cancer and stereotactic radiosurgery (SRS) (cohort)

Primary cancer sites	No. of patients	Presentation		Interval (months) between diagnosis of primary cancer and SRS	
		Synchronous	Metachronous	Mean/median	Maximum/IQR
Lung	1840	378 (20.5 %)	1462 (79.5 %)	17/10	262/3–21
Breast	309	4 (1.3 %)	305 (98.7 %)	59/48	289/27–78
GI tract	328	25 (7.6 %)	303 (92.4 %)	34/24	237/11–45
Kidney	115	10 (8.7 %)	105 (91.3 %)	55/29	324/8–90

*GI* gastro-intestinal

p < 0.0001

In contrast, Sanchez et al. found neurologically asymptomatic NSCLC patients to have longer survival; the MST of 12 asymptomatic patients was 7.5 months and that of 69 symptomatic patients was 4.0 months (p = 0.02) [34]. Moreover, they also reported that control of the neurological status of asymptomatic patients is better than that of symptomatic patients (80 vs. 40 %) [34]. However, these reports described a small number of patients. Yamamoto, Serizawa, and colleagues reported that, based on 1196 prospectively enrolled patients undergoing gamma knife SRS, there were significant MST differences in Mets between asymptomatic and symptomatic patient groups (HR 1.779, 95 % CI 1.541–1.589, p < 0.0001) [35]. However, in both reports, there were considerable biases between the asymptomatic and symptomatic patient groups, i.e., the tumor volume was larger and KPS scores were lower in the symptomatic than in the asymptomatic group [34].

The major weakness of the present study is that it was retrospective. As we discussed in our previous articles, the characteristics of patients receiving a particular treatment regimen are considered to have a major influence on treatment selection. This is an important issue when estimating the effects of treatments or exposures on outcomes using observational data. One approach to reducing or eliminating the effect of treatment selection bias and confounding effects is to use propensity score matching, which allows one to design and analyze an observational (non-randomized) study that mimics some of the characteristics of a randomized controlled trial. Therefore, in the present investigation, a case-matched study was also conducted by one of the authors (Y.S.), who did not participate in other aspects of this study and was blinded to final outcomes. Nevertheless, because this was a retrospective study, even with the application of case matching, biases in patient selection, original cancer treatments over time, follow-up (outcome, toxicity, and imaging), observers, and so on, could not be eliminated.

The other weakness of this study is the lack of information on tumor locations. It is absolutely crucial to describe tumor location when neurological symptoms are discussed. However, unfortunately, our retrospectively-accumulated database did not include information on tumor locations and, therefore, we could not examine the impact of this factor.

Moreover, there are several differences in welfare and health insurance systems between Japan and other countries, and these have major impacts on the cost of screening examinations such as MRI. Factoring in quality-adjusted life years for patients receiving versus not receiving routine scanning would be useful.

A randomized controlled trial, in the near future, is necessary to test the hypothesis put forth herein.

## Conclusion

Neurologically asymptomatic patients who underwent SRS for BM had better results than symptomatic patients in terms of maintenance of a good neurological state and a reduction in the neurological death rate. Our results indicate that screening for BM by CT/MRI may be beneficial for managing cancer patients. These observations merit further detailed research.
